# Effects of Mung Bean Water Supplementation on Modulating Lipid and Glucose Metabolism in a Diabetic Rat Model

**DOI:** 10.3390/nu16162684

**Published:** 2024-08-13

**Authors:** Chung-Hsiung Huang, Jia-Yin Chen, Meng-Tsan Chiang

**Affiliations:** Department of Food Science, National Taiwan Ocean University, Keelung 20224, Taiwan; huangch@mail.ntou.edu.tw (C.-H.H.); joy-03-21@yahoo.com.tw (J.-Y.C.)

**Keywords:** diabetes mellitus, mung bean water, dietary supplement, hypoglycemia, hypolipidemia, carbohydrate metabolism, lipid metabolism

## Abstract

Type 2 diabetes mellitus (T2DM) is often associated with chronic inflammation exacerbated by hyperglycemia and dyslipidemia. Mung beans have a longstanding reputation in traditional medicine for their purported ability to lower blood glucose levels, prompting interest in their pharmacological properties. This study aimed to explore the impact of mung bean water (MBW) on carbohydrate and lipid metabolism in a T2DM rat model induced by nicotinamide/streptozotocin. Normal and DM rats were supplemented with a stock solution of MBW as drinking water ad libitum daily for 8 weeks. MBW supplementation led to significant reductions in plasma total cholesterol, HDL-C, and VLDL-C + LDL-C levels, and decreased malondialdehyde levels in plasma and liver samples, indicating reduced oxidative stress. MBW supplementation lowered plasma glucose levels and upregulated hepatic hexokinase activity, suggesting enhanced glucose utilization. Additionally, MBW decreased hepatic glucose-6-phosphate dehydrogenase and glutathione peroxidase activities, while hepatic levels of glutathione and glutathione disulfide remained unchanged. These findings underscore the potential of MBW to improve plasma glucose and lipid metabolism in DM rats, likely mediated by antioxidant effects and the modulation of hepatic enzyme activities. Further exploration of bioactive components of MBW and its mechanisms could unveil new therapeutic avenues for managing diabetes and its metabolic complications.

## 1. Introduction

Type 2 diabetes mellitus (T2DM) is characterized by insulin resistance and impaired glucose regulation, presenting significant metabolic challenges. The oral glucose tolerance test (OGTT) is essential for diagnosing T2DM, evaluating the ability of the host to process glucose over time [1]. Parameters such as drinking behavior, urine volume, food intake, feces weight, body weight, and tissue weights are vital for assessing metabolic homeostasis and dysregulation in diabetic conditions. In individuals with T2DM, disturbances in plasma glucose levels, insulin secretion, and markers like fructosamine reflect the severity of insulin resistance and glycemic control.

The nicotinamide/streptozotocin (STZ)-induced T2DM rat model is widely used in research to simulate the pathophysiology of human T2DM. This model combines nicotinamide, a precursor of nicotinamide adenine dinucleotide, with STZ, a naturally occurring glucosamine-nitrosourea compound, to induce selective pancreatic β-cell toxicity and insulin resistance [2]. The sequential administration of nicotinamide and STZ effectively replicates key aspects of T2DM, including β-cell dysfunction, insulin resistance, hyperglycemia, and dyslipidemia, resembling the progressive nature of the disease in humans [2]. Key features of the nicotinamide/STZ-induced T2DM rat model include its reproducibility, feasibility, and ability to mimic several metabolic abnormalities observed in human T2DM. These include altered plasma glucose and insulin levels, dysregulated lipid metabolism, oxidative stress markers, hepatic dysfunction, and changes in antioxidant enzyme activities [3]. Moreover, this model allows for the investigation of therapeutic interventions aimed at ameliorating β-cell dysfunction, improving insulin sensitivity, and attenuating diabetic complications [4].

Mung bean water (MBW), derived from soaking mung beans in water, has gained attention for its potential health benefits. Mung beans (*Vigna radiata* L.) are rich in bioactive compounds such as polyphenols, flavonoids, and peptides, known for their antioxidant and anti-inflammatory properties [5,6,7,8]. Mung bean extract contains these bioactive components that contribute to its therapeutic effects. Studies have shown that mung bean extract exhibits hypoglycemic effects by improving insulin sensitivity and regulating glucose metabolism [9,10]. It has also been reported to lower fasting blood glucose levels and improve glucose tolerance. Additionally, the consumption of mung bean extract has been associated with reductions in plasma cholesterol, triglycerides, and oxidative stress markers in high-fat-diet-induced obese mice [6,11]. Furthermore, mung bean extract has demonstrated hepatoprotective effects by enhancing antioxidant enzyme activities and reducing lipid peroxidation in the liver [12,13]. These effects are crucial in mitigating hepatic complications associated with T2DM and improving liver function. The bioactive components present in mung bean extract not only address metabolic abnormalities but also exert anti-inflammatory effects, which are beneficial in preventing chronic complications associated with T2DM [14,15]. Therefore, it is hypothesized that MBW represents a promising dietary intervention for managing T2DM due to its multifaceted biological activities. While the antidiabetic potential of mung bean extract and its derived components, such as proteins, peptides, and polyphenols, has been reported, the modulatory effects of MBW on glucose and lipid metabolism, as well as its antioxidant activity in normal and T2DM rats, remain unclear [9,11,16,17,18,19,20]. In this study, a comprehensive investigation was conducted to understand the influence of MBW on carbohydrate and lipid metabolism and anti-oxidative capacity in normal rats and nicotinamide/STZ-induced T2DM rats.

## 2. Materials and Methods

### 2.1. Preparation of Mung Bean Water (MBW)

Mung beans (*Vigna radiata* (L.) Wilczek), which were cultured and harvested in Puzi, Chiayi, Taiwan, were purchased from Rongfeng rice store. After stirring 1.5 kg of mung beans with 10 L of distilled water at 7 °C in a cold room for 14 h, the supernatant was collected as the stock solution of MBW. Part of the stock solution of MBW was concentrated and lyophilized for component analysis following the methods reported in previous studies [21,22]. The yield of freeze-dried MBW powder was 0.09%, and the lyophilized MBW powder contained 7.11% gallic acid-equivalent total polyphenolic compounds, 4.50% crude protein, 8.31% moisture, and 0.16% crude fat.

### 2.2. Animals and Experimental Diets

Ten-week-old male Sprague Dawley rats were obtained from the National Laboratory Animal Center of Taiwan. For acclimatization, all rats were housed in disinfected stainless steel cages maintained at a temperature of 23 ± 1 °C, a humidity of 40–60%, and a light cycle of 12 h per day for 3 weeks, and were fed solid feed consisting of 20% casein, 10% lard, 1% vitamin mixture (AIN 76 vitamin mixture), 4% salt mixture (AIN 76 mineral mixture), 0.5% cholesterol, 0.3% cholic acid, 0.2% choline chloride, 5% cellulose, and 59% corn starch. Feed and distilled water were provided ad libitum. After acclimatization, T2DM was induced in rats with body weights of more than 300 g via subcutaneous injection of nicotinamide (230 mg/kg body weight) and STZ (65 mg/kg body weight in citrate buffer, pH 4.6). An OGTT was conducted to determine whether the nicotinamide/streptozotocin injection successfully induced diabetes. The time point 0 min was established by promptly collecting blood samples for glucose detection immediately after glucose administration. One week after nicotinamide and STZ injection, an OGTT was performed. The rats were randomly divided into four groups: normal rats given distilled water, normal rats given the stock solution of MBW as drinking water, diabetic rats given distilled water, and diabetic rats given the stock solution of MBW as drinking water. Each group contained 8–10 rats, and the rats were supplemented with either water or MBW ad libitum for 8 weeks. Three days before euthanasia, food intake, water consumption, defecation, and urine output were measured, and feces samples were collected. Moreover, the OGTT was conducted again, and the area under the curve (AUC) and delta area under the curve (ΔAUC) were calculated using the formula described in a previous study [23]. AUC (mg × h/dL) = plasma glucose at 0 min + 2 × plasma glucose at 30 min + 2 × plasma glucose at 60 min + 2 × plasma glucose at 120 min + plasma glucose at 180 min/4. ΔAUC (mg × h/dL) = (plasma glucose at 0 min − plasma glucose at 0 min) + 2 × (plasma glucose at 30 min − plasma glucose at 0 min) + 2 × (plasma glucose at 60 min − plasma glucose at 0 min) + 2 × (plasma glucose at 120 min − plasma glucose at 0 min) + (plasma glucose at 180 min − plasma glucose at 0 min)/4. On the day of euthanasia, rats were anesthetized with isoflurane, and the blood, heart, liver, kidney, epididymal fat, and adrenal fat tissues were collected for further analysis.

### 2.3. Determination of Plasma Lipids, Glucose, Insulin, Frutosamine, Leptin, Lactate, and Transaminase

The levels of total cholesterol, free fatty acid, triglyceride, glucose, insulin, fructosamine, leptin, lactate, alanine transaminase (ALT), and aspartate transaminase (AST) were analyzed using commercially available kits (Sigma Co., St. Louis, MO, USA; Assay Designs, Inc., Ann Arbor, MI, USA; BioVision Research Products, Mountain View, CA, USA; Audit Diagnostics, Cork, Ireland). According to the characteristics of differing lipoprotein density, the ultracentrifugation method was used to separate and analyze the levels of lipoproteins in the plasma as previously described [24]. A Hitachi CP90NX ultracentrifuge (Tokyo, Japan) was used to segregate high-density lipoprotein-cholesterol (HDL-C), low-density lipoprotein-cholesterol (LDL-C) and very low-density lipoprotein-cholesterol (VLDL-C) in the plasma by means of density gradient ultracentrifugation (194,000× *g* at 10 °C for 3 h). The HDL-C, LDL-C, and VLDL-C were then recovered, and the cholesterol in the various separated lipoproteins was measured using the aforementioned enzymatic methods.

### 2.4. Determination of Hepatic and Fecal Lipids

According to the method of Folch et al., liver or fecal samples were homogenized with a solution consisting of 20 times its volume of chloroform/methanol mixture (2:1; *v*/*v*) using a homogenizer (Hong Sheng, SA-50 Max. 3000, Taipei, Taiwan) [25]. After homogenization, the mixture was filtered through filter paper, and the clear filtrate was then concentrated using a vacuum concentrator (Savant, Speed Vac. SC 110, 1725 rpm, Farmingdale, NY, USA) to remove organic solvents. The residue was then adjusted to a volume of 10 mL with the chloroform/methanol mixed solution (2:1; *v*/*v*) and stored in a vial for lipid analysis. The contents of lipids in liver or fecal samples were measured using the aforementioned enzymatic methods.

### 2.5. Determination of TBARSs and Hepatic GSH, GSSG, Hexokinase, G-6-Pase, and G-6-P DeHase Activities

The measurement of thiobarbituric acid reactive substances (TBARSs) was performed by means of the reaction between thiobarbituric acid and a lipid peroxide product (malondialdehyde, MDA) in the samples of plasma and liver lysate. 1,1,3,3-tetraethoxypropane (Sigma-Aldrich, St. Louis, MO, USA) was used as a standard and saline was used as a blank. The solutions underwent a 45 min incubation in a boiled water bath, and then were allowed to cool. Following centrifugation at 1600× *g* and 4 °C for 10 min, the resulting supernatants were incubated at room temperature for 30 min. Subsequently, MDA levels were measured using a Synergy HT microplate reader (BioTek, Winooski, VT, USA) with excitation at 515 nm and emission at 553 nm.

The methods used to determine glutathione (GSH), glutathione disulfide (GSSG), and glutathione peroxidase (GSH Px) were those outlined in [26]. Briefly, the liver samples were homogenized with 1% picric acid and then centrifuged at 10,000× *g* for 20 min. From the obtained supernatant, 5 µL was used to measure total GSH. On the other hand, 100 µL of the supernatant was mixed with 2 µL of 2-vinylpyridine and allowed to react for 60 min, after which 5 µL was used for GSSG measurement. For both samples, 5 µL was added to 0.7 mL of 0.2 mM NADPH buffer, 0.1 mL of 0.6 mM DTNB, and 195 µL of distilled water, and the mixture was incubated at a constant temperature of 30 °C for 4 min. Finally, 5 µL of 200-unit GSH reductase solution was added, and the absorbance was measured at 412 nm for 3 min. The liver GSH content was determined by comparing with standard samples.

To assess the function of hepatic enzymes involved in regulating the internal glucose balance, liver samples were processed by means of homogenization in N-acetyl-cysteine buffer followed by centrifugation to isolate hepatocyte cytosol. Activities of hexokinase, glucose-6-phosphatase (G-6-Pase), and glucose-6-phosphate dehydrogenase (G-6-P DeHase) in the cytosolic fraction were determined using the established methods [27].

### 2.6. Statistical Analysis

The experimental data were analyzed using the SPSS/PC v28 statistical analysis software. Differences between each experimental group and its control group were assessed using independent-sample *t*-tests. The effects of DM and MBW consumption were analyzed using two-way analysis of variance (ANOVA), examining the individual effects of these two factors and their interaction.

## 3. Results

### 3.1. Impact of MBW Supplementation on Plasma Glucose Levels and Body/Organ Weights in Normal and Diabetic Rats

One week after nicotinamide/STZ injection, an OGTT was conducted. Plasma glucose concentrations of DM rats at 30, 60, and 120 min showed significant increases compared to normal rats (Table 1). The peak blood glucose concentration in the DM rats was close to 200 mg/dL, and the time taken to return to the initial glucose concentration was longer compared to the normal rats (Table 1). Another OGTT was conducted after 8 weeks of MBW supplementation. Plasma glucose concentrations of DM rats at each time point were significantly higher compared to normal rats. Rats supplemented with MBW showed a significant decrease in plasma glucose at 0 and 120 min compared to rats supplemented with distilled water. At 120 and 180 min, both DM and MBW factors had an effect on plasma glucose (Table 1). Notably, the values of AUC and ΔAUC in DM rats were elevated compared to those in normal rats. Rats supplemented with MBW showed lower AUC values than those supplemented with water, although there was no statistically significant difference (Table 1).

During the 8-week supplementation period, there was no statistically significant difference in body weight between rats supplemented with MBW and those supplemented with distilled water (Table 2). In normal rats, the group supplemented with MBW showed a significant increase in water intake compared to the group supplemented with distilled water. In DM rats, feeding with MBW significantly increased water intake. Additionally, there were no statistically significant differences in food intake, urine volume, and fecal volume between the groups of DM and normal rats (Table 2). On the other hand, the liver weight and liver-to-body weight ratio of DM rats were significantly higher than those of normal rats. Feeding normal rats with MBW significantly increased kidney weight, but there was no significant difference in kidney weight per 100 g of body weight between the two groups of normal rats. Additionally, there were no statistically significant differences in white adipose tissue weight between the DM and normal groups (Table 2).

### 3.2. Hypoglycemic and Hypolipidemic Effects of MBW Supplementation

After feeding for 8 weeks, the plasma glucose concentrations of DM rats were significantly higher than those of normal rats. DM rats supplemented with MBW had significantly lower plasma glucose concentrations compared to DM rats supplemented with distilled water (Table 3). There was no statistically significant difference in the plasma insulin concentrations among the four groups (Table 3). It has been reported that elevated fructosamine levels indicate prolonged hyperglycemia, while the OGTT provides insights into insulin sensitivity and resistance [28]. Notably, the interaction between the factors DM and MBW had an effect on plasma frutosamine in each group of rats, while feeding with MBW significantly decreased plasma fructosamine concentrations in DM rats (Table 3). There were no statistically significant differences in plasma leptin concentrations among the four groups (Table 3). The plasma lactate concentration of DM rats was significantly higher than that of normal rats. Regardless of whether they were normal or DM rats, feeding with MBW significantly increased plasma lactate concentrations (Table 3). The protocol for T2DM induction followed that described in previous studies [29,30]. As shown in Table 2 and Table 3, rats injected with nicotinamide/STZ had significantly higher plasma glucose levels in the OGTT and higher fasting glucose levels. Additionally, there were no statistically significant differences in plasma insulin concentrations among the four groups. Therefore, the nicotinamide/STZ injection induced insulin-resistant diabetes (T2DM) in the rats.

Altered lipid metabolism in T2DM contributes significantly to cardiovascular risks. Dyslipidemia, characterized by elevated levels of VLDL-C and LDL-C and reduced levels of HDL-C, exacerbates atherosclerosis and cardiovascular complications [31]. In the current study, feeding with MBW significantly reduced the concentrations of plasma free fatty acids in DM rats (Table 3). Compared to feeding with distilled water, feeding with MBW significantly reduced the concentrations of total cholesterol in the plasma of both normal and DM rats (Table 3). There were no significant differences in plasma triglyceride concentrations among the four groups (Table 3). Feeding with MBW significantly reduced the concentrations of VLDL-C + LDL-C and HDL-C in the plasma of DM rats (Table 3). The ratio of plasma total cholesterol to HDL-C in DM rats significantly increased, but feeding with MBW had no significant effect on it (Table 3).

### 3.3. Influences of MBW Supplementation on Modulating Lipid Metabolism and Glucose Metabolism-Related Enzymes

The levels of cholesterol and triglycerides per gram of liver tissue, as well as the levels of cholesterol and triglycerides in the liver, were significantly increased in DM rats compared to those in normal rats (Table 4). DM rats supplemented with MBW showed significantly decreased contents of cholesterol and triglycerides per gram of liver tissue compared to DM rats supplemented with distilled water (Table 4). Feeding with MBW significantly reduced the contents of total triglycerides and triglycerides in the liver per gram in normal rats (Table 4).

Hepatic enzymes such as hexokinase, G-6-Pase, and gG-6-P DeHase play crucial roles in glucose metabolism and are dysregulated in T2DM. Hexokinase initiates glycolysis by phosphorylating glucose, whereas G-6-Pase catalyzes the final step of gluconeogenesis, contributing to elevated blood glucose levels. G-6-P DeHase participates in the pentose phosphate pathway, influencing antioxidant defense mechanisms through NADPH production [32]. Regarding the expression of hepatic glucose metabolism-related enzymes, the interaction between the factors DM and MBW affected the activity of hexokinase in each group, and feeding with MBW significantly increased the activity of hexokinase in the liver (Table 4). However, there was no statistically significant difference in G-6-Pase activity and the ratio of hexokinase/G-6-Pase among the four groups (Table 4). Notably, MBW supplementation significantly reduced the activity of G-6-P DeHase in the liver of DM rats (Table 4).

The interaction between the factors DM and MBW affected the total cholesterol concentration per gram of feces in each group of rats, and feeding with MBW significantly reduced the cholesterol concentration per gram of feces in both normal and DM rats (Table 5). Nevertheless, there were no statistically significant differences in triglyceride concentration in feces among the groups (Table 5).

### 3.4. Antioxidative Activity of MBW

Antioxidant markers such as GSH, GSSG, and GSH Px reflect the oxidative stress levels in T2DM. GSH, a key antioxidant, maintains redox balance, whereas GSH Px enzymes protect against lipid peroxidation and cellular damage [33]. TBARSs serve as biomarkers of lipid peroxidation, elevated in conditions of oxidative stress such as T2DM. Monitoring TBARS levels provides insights into oxidative damage progression and cellular health [34]. The level of GSH in the liver of DM rats was significantly reduced compared to normal rats, while there were no statistically significant differences in GSSG expression among the four groups (Table 6). The interaction between the factors DM and MBW affected the hepatic level of GSH Px, and feeding with MBW significantly increased the activity of GSH Px in the livers of DM rats (Table 6). Moreover, the interaction between the factors DM and MBW affected the plasma TBARS contents, and feeding with MBW significantly reduced the expression of TBARSs in the plasma of both normal and DM rats (Table 6). In parallel, feeding with MBW significantly reduced the expression of hepatic TBARSs of DM rats but not affected nephrotic TBARS expression (Table 6). ALT and AST are plasma enzymes indicating hepatocellular injury and metabolic stress in T2DM [35]. Elevated ALT and AST levels correlate with liver dysfunction and increased risk of liver disease. Understanding the interplay of these biochemical and physiological parameters in T2DM is crucial for developing targeted therapeutic strategies to improve metabolic outcomes and mitigate diabetes-related complications. Remarkably, there was no statistically significant difference in the activity of plasma AST and ALT among the four groups (Table 6).

## 4. Discussion

The therapeutic potential of mung bean and its derivatives in managing metabolic disorders has been highlighted through several studies involving different animal or cell models. Our findings are consistent with previous research showing that mung bean interventions can significantly impact glucose metabolism and related parameters. It has been demonstrated that the oral administration of mung bean sprout extracts (2 g/kg) and mung bean seed coat extracts (3 g/kg) to KK-Ay mice over 5 weeks led to reductions in blood glucose, plasma C-peptide, glucagon, total cholesterol, and triglyceride levels. These interventions also markedly improved glucose tolerance and increased insulin immunoreactive levels. These results support the potential of mung bean extracts in ameliorating multiple facets of metabolic syndrome [9]. In another study, the oral administration of ethanol extracts of mung bean testa to diabetic KK-Ay mice for 4 weeks resulted in a decrease in the total weight of white adipose tissue, triacylglycerol, and total cholesterol levels in muscle. Additionally, it suppressed the expression of lipogenic genes and reduced the plasma levels of pro-inflammatory cytokines. This evidence underscores the role of mung bean testa in modulating lipid metabolism and inflammation [11]. Further research with mung bean water extract revealed that it enhanced cellular glucose uptake and modified the expression of glucose metabolism-related genes in insulin-resistant HepG2 cells. This extract also inhibited both α-amylase and α-glucosidase and prevented the formation of advanced glycation end products. These results indicate that mung bean water extract could improve insulin sensitivity and glucose metabolism through multiple mechanisms, including PTP-1B inhibition [19]. A 5-week dietary intervention with mung bean peptide (245 mg/kg/day) in prediabetic mice induced by a high-fat diet resulted in significant reductions in body weight gain, hyperglycemia, hyperlipidemia, insulin resistance, inflammation, and oxidative stress. Additionally, this intervention alleviated liver and kidney damage and reversed gut microbiota imbalance. This impact highlights the potential of mung bean peptide in addressing various metabolic disturbances associated with obesity and prediabetes [17]. Similarly, a 5-week dietary intervention with mung bean peptide (245 mg/kg/day) in high-fat-diet-treated mice demonstrated notable improvements in insulin resistance, body weight, and several serum biomarkers including fasting blood glucose, C-peptide, IL-6, TNF-α, and MDA. The treatment also increased superoxide dismutase content, enhanced pancreatic β-cell function, and repaired damaged pancreatic tissue. These findings suggest that mung bean peptide may regulate several metabolic pathways, including amino acid, glycerol phospholipid, fatty acid, alkaloid, and nicotinamide metabolism, to improve the overall metabolic profile of high-fat diet mice [18]. Additionally, a dietary intervention with polyphenol extract from germinated mung beans (50–150 mg/kg) in diabetic C57BL/6 mice for 5 weeks resulted in decreased fasting blood glucose, insulin resistance, and serum levels of ALT and pro-inflammatory cytokines. It also improved glucose tolerance, serum lipid indexes, liver tissue morphology, and intestinal flora imbalances. This indicates that polyphenol-rich extracts from mung beans can enhance metabolic health and address systemic inflammation and dysbiosis in diabetic conditions [20]. Recently, a 4-week intervention with a combination drink of mung bean extract and ginger extract was found to be effective in lowering post-prandial blood glucose levels in T2DM rats. This finding suggests that the combination of these extracts may offer a promising approach for glycemic control in diabetic conditions [16]. In the current study, the rats received 33.6–64.8 mL of MBW per day (Table 3), which is approximately equivalent to a dose of 60–120 mg/kg of lyophilized MBW powder, considering that the yield of freeze-dried MBW powder was 0.09%. Notably, the lyophilized MBW powder contained 7.11% gallic acid equivalent total polyphenolic compounds, 4.50% crude protein, 8.31% moisture, and 0.16% crude fat. Consequently, it was calculated that each rat received 4.3–8.5 mg/kg of total polyphenolic compounds, 2.7–5.4 mg/kg of protein, and 0.1–0.19 mg/kg of fat from MBW per day.

In this study, DM and normal rats, regardless of whether they were supplemented with MBW or distilled water, did not exhibit significant differences in their body weight, adipose tissue mass, lipid-to-body weight ratios, or kidney-to-body weight ratios. However, DM rats showed significantly higher liver weights and liver-to-body weight ratios compared to normal rats. This finding aligns with previous research indicating that STZ-induced diabetic rats often exhibit an increased liver weight due to enhanced lipid accumulation in the liver [36]. Insulin resistance in diabetes triggers adipose tissue lipolysis, elevating the levels of plasma free fatty acids that are subsequently stored as triglycerides in the liver, contributing to increased liver weight and lipid content [37]. Consistently, our study found DM rats to have significantly elevated total cholesterol and triglyceride levels in the liver compared to normal rats, suggesting a correlation between liver weight increase and lipid accumulation in diabetes. Despite no significant differences in food intake, urine volume, or feces volume among groups, rats supplemented with MBW consumed more water compared to those given regular water. This could be attributed to the aromatic compounds present in MBW, known to enhance palatability and increase water consumption [38]. On the other hand, damage to pancreatic β-cells by STZ leads to reduced insulin secretion, contributing to elevated blood glucose levels in diabetic rats [39]. While our study did not find significant differences in plasma insulin concentrations between DM and normal rats, MBW effectively reduced plasma glucose levels in DM rats, potentially through mechanisms enhancing hepatic hexokinase activity. Hexokinase facilitates glucose metabolism by converting glucose to G-6-Pase, a key step in glycolysis inhibited by insulin resistance [40]. Previous studies have shown that feeding rats with rutin for 45 days or pterostilbene for 42 days significantly increased hexokinase enzyme activity in diabetic rat livers. Rutin and pterostilbene increased insulin secretion in diabetic rats, promoting glycolysis [29,39]. In contrast, it has been reported that feeding rats with genistein for 21 days or rutin for 45 days significantly inhibited G-6-Pase activity in diabetic rats. The reduced insulin secretion in STZ-induced diabetic rats led to increased expression of G-6-Pase mRNA, which was countered by genistein and rutin [39,40]. In this study, supplementing DM rats with MBW significantly increased hepatic hexokinase activity, while there was no significant difference in G-6-Pase activity among the groups. It is suggested that polyphenolic compounds in MBW may promote glycolysis by increasing hexokinase activity, thereby lowering plasma glucose levels.

In the current study, DM rats supplemented with MBW exhibited significantly lower plasma concentrations of total cholesterol, HDL-C, and VLDL-C + LDL-C compared to those supplemented with distilled water. This reduction in plasma total cholesterol is likely attributed to the ability of MBW to effectively lower VLDL-C + LDL-C levels in DM rats. Although HDL-C also showed a decrease with MBW supplementation, the ratio of total cholesterol to HDL-C did not significantly differ between groups. Plasma free fatty acids are key contributors to hepatic lipid accumulation. Elevated plasma free fatty acid levels lead to an increase in their transport to the liver, where they are either stored as triglycerides or metabolized into acetyl-CoA, contributing to cholesterol synthesis [37]. Increased plasma free fatty acid levels are associated with insulin resistance, impairing glucose transport into cells [41]. Our findings indicate that MBW significantly reduced plasma free fatty acid concentrations in DM rats, suggesting a potential improvement in insulin sensitivity. In terms of liver lipid content, DM rats supplemented with MBW showed reduced cholesterol levels compared to those supplemented with distilled water. Previous studies suggest that compounds like genistein and daidzein can inhibit liver fatty acid synthase and β-oxidation enzymes, reducing acetyl-CoA production and lowering cholesterol and triglyceride levels in the liver [42]. Similarly, MBW may influence these pathways, thereby reducing liver lipid content in diabetic rats. Interestingly, while there were no significant differences in fecal triglyceride content among the groups, DM rats supplemented with MBW exhibited a significant decrease in fecal cholesterol content. Further investigations are necessary to elucidate the mechanisms behind this observation. Moreover, the antioxidative properties of MBW were also evident in our study, as indicated by reduced plasma and hepatic TBARs concentrations in DM rats. This suggests that polyphenolic compounds contained in MBW may scavenge free radicals, thereby reducing lipid peroxide levels and mitigating oxidative stress. These findings highlight the potential of MBW in improving lipid profiles and reducing liver lipid accumulation in DM rats. Future studies should focus on identifying the specific bioactive compounds responsible for these effects.

Hyperglycemia in diabetes exacerbates oxidative stress by increasing the production of reactive oxygen species, which impair β-cell function [30]. STZ, used to induce diabetes in animal models, stimulates pancreatic H_2_O_2_ production, damaging β-cell DNA and escalating oxidative stress [30]. This study demonstrates that MBW effectively reduced plasma and hepatic TBARS production in both normal and DM rats. The antioxidative properties of MBW are likely attributable to its polyphenolic compounds, which act as electron donors and free radical scavengers, thereby reducing lipid peroxide levels in diabetic tissues [38,43]. DM rats supplemented with MBW could take in greater amounts of polyphenolic compounds, which contributed to reductions in the levels of oxidative stress markers in the plasma and liver tissues. Previous research has shown that substances like genistein and quercetin can increase GSH Px activity in diabetic rats, enhancing antioxidant capacity [40,44]. In contrast, our study found decreased G-6-P DeHase and GSH Px activities in DM rats supplemented with MBW. This disparity may be explained by differences in oxidative stress levels between the employed diabetic animal model, which received nicotinamide prior to STZ injection for pancreatic protection, and previous models inducing severe oxidative stress via STZ alone. Moreover, reductions in liver GSH Px and G-6-P DeHase activities suggest a modulation of NADPH-dependent oxidative pathways. These findings underscore the potential of MBW as a dietary supplement to mitigate oxidative stress and enhance antioxidant defenses in diabetes, warranting further investigation into its mechanisms and clinical applications.

## 5. Conclusions

The findings from this study demonstrate the beneficial effects of MBW on DM rats over an 8-week period. Significant reductions were observed in fasting plasma glucose, cholesterol, and free fatty acids. Moreover, MBW effectively lowered both plasma and hepatic TBARs levels in DM rats, while also reducing liver enzyme activities associated with glucose metabolism. These results suggest that MBW holds promise as a natural intervention for managing metabolic abnormalities in diabetes. 

## Figures and Tables

**Table 1:** Results of OGTTs before and after an 8-week MBW supplementation period.

Ingredient ^1^	DM		Normal		Two Way ANOVA ^1^		
	Water	MBW	Water	MBW	DM	MBW	DM × MBW
Before treatment							
0 min	110.29 ± 12.34	107.24 ± 14.17	103.45 ± 20.28	107.43 ± 18.50	N.S. ^2^	N.S.	N.S.
30 min	188.22 ± 39.44	191.48 ± 35.95	156.44 ± 28.02	175.21 ± 18.53	0.025	N.S.	N.S.
60 min	179.56 ± 33.00	212.65 ± 45.07	143.86 ± 21.10	141.19 ± 22.17	0.000	N.S.	N.S.
120 min	133.58 ± 11.47	155.81 ± 32.73	128.88 ± 11.38	132.52 ± 19.97	0.045	0.025	N.S.
180 min	124.76 ± 23.90	129.63 ± 29.45	129.01 ± 21.45	103.68 ± 12.72	N.S.	N.S.	0.046
After treatment for 8 weeks							
0 min	140.94 ± 25.37	126.29 ± 26.25	126.57 ± 22.16	109.62 ± 11.69 *	0.035	0.033	N.S.
30 min	277.34 ± 84.08	254.31 ± 32.05	180.57 ± 36.92	179.30 ± 24.89	0.000	N.S.	N.S.
60 min	210.25 ± 45.24	203.41 ± 36.35	177.20 ± 31.39	156.07 ± 8.10 *	0.001	N.S.	N.S.
120 min	180.19 ± 36.99	184.72 ± 28.68	173.12 ± 25.06	133.49 ± 10.70 *	0.003	0.066	0.022
180 min	179.46 ± 23.43	196.27 ± 36.40	163.46 ± 24.04	138.49 ± 26.25 *	0.000	N.S.	N.S.
AUC	1521.35 ± 324.36	1438.72 ± 192.85	1208.45 ± 127.16	1156.07 ± 85.96	0.000	N.S.	N.S.
ΔAUC	499.53 ± 298.45	531.86 ± 248.72	283.01 ± 99.94	348.38 ± 115.66	N.S.	N.S.	N.S.

Results are expressed as mean ± standard deviation for 8–10 rats per group. * *p* < 0.05 compared with the respective control via the unpaired *t*-test. ^1^ DM stands for diabetes mellitus; MBW stands for mung bean water; DM × MBW represents the diabetes and mung bean water interaction assessed by two-way ANOVA. ^2^ N.S. stands for not significant.

**Table 2:** Levels of drinking, urine volume, feed intake, feces weight, body weight, and tissue weights in rats supplemented with MBW for 8 weeks.

Ingredient ^1^	DM		Normal		Two Way ANOVA ^1^		
	Water	MBW	Water	MBW	DM	MBW	DM × MBW
Drinking volume (mL)	38.5 ± 8.31	49.2 ± 15.6 *	41.5 ± 13.8	48.1 ± 10.5	N.S. ^2^	0.043	N.S.
Urine volume (mL)	19.8 ± 3.98	23.3 ± 8.43	23.7 ± 11.7	27.8 ± 5.52	N.S.	N.S.	N.S.
Feed intake (g)	25.5 ± 3.87	25.6 ± 2.35	27.4 ± 4.96	25.2 ± 4.59	N.S.	N.S.	N.S.
Feces (g)	2.10 ± 0.21	2.02 ± 0.19	1.99 ± 0.17	2.04 ± 0.28	N.S.	N.S.	N.S.
Initial body weight (g)	460.7 ± 35.4	435.9 ± 27.3	474.8 ± 24.2	474.8 ± 31.8	N.S.	N.S.	N.S.
Final body weight (g)	644.4 ± 56.1	639.7 ± 72.6	671.7 ± 42.6	650.7 ± 50.3	N.S.	N.S.	N.S.
Liver weight (g)	37.1 ± 6.57	36.7 ± 5.41	32.7 ± 4.26	31.8 ± 4.59	0.009	N.S.	N.S.
Liver weight (g)/100 g body weight	5.89 ± 0.80	5.83 ± 0.63	5.01 ± 0.43	5.04 ± 0.54	0.000	N.S.	N.S.
Kidney weight (g)	3.53 ± 0.59	3.49 ± 0.28	3.54 ± 0.29	3.18 ± 0.36 *	N.S.	N.S.	N.S.
Kidney weight (g)/100 g body weight	0.56 ± 0.07	0.54 ± 0.04	0.54 ± 0.04	0.52 ± 0.05	N.S.	N.S.	N.S.
Adipose tissue weight (g)	13.0 ± 2.62	12.8 ± 5.11	14.4 ± 4.23	13.3 ± 2.84	N.S.	N.S.	N.S.
Adipose tissue weight (g)/100g body weight	2.06 ± 0.26	2.14 ± 0.48	2.19 ± 0.58	2.11 ± 0.37	N.S.	N.S.	N.S.

Results are expressed as mean ± standard deviation for 8–10 rats per group. * *p* < 0.05 compared with the respective control via the unpaired *t*-test. ^1^ DM stands for diabetes mellitus; MBW stands for mung bean water; DM × MBW represents the diabetes and mung bean water interaction assessed by two-way ANOVA. ^2^ N.S. stands for not significant.

**Table 3:** Plasma levels of glucose, insulin, fructosamine, and lipids in rats supplemented with MBW for 8 weeks.

	DM		Normal		Two Way ANOVA ^1^		
	Water	MBW	Water	MBW	DM	MBW	DM × MBW
Glucose (mg/dL)	159.7± 32.3	121.7 ± 29.1 *	129.6 ± 17.9	126.4 ± 12.4	0.04	0.017	N.S.
Insulin (μg/L)	1.13 ± 0.91	1.31 ± 0.75	1.41 ± 0.64	1.93 ± 0.98	N.S. ^2^	N.S.	N.S.
Frutosamine (μmol/L)	464.4 ± 189.6	190.7 ± 84.3 *	197.1 ± 51.6	172.3 ± 54.8	0.000	0.000	0.002
Leptin (pg/L)	3.34 ± 0.94	2.62 ± 0.94	3.06 ± 1.22	2.74 ± 0.76	N.S.	N.S.	N.S.
Lactate (mg/dL)	28.7 ± 8.5	31.5 ± 7.8	19.1 ± 4.7	26.0 ± 4.8 *	0.001	0.030	N.S.
Free fatty acid (mEq/L)	0.47 ± 0.05	0.41 ± 0.05 *	0.43 ± 0.14	0.43 ± 0.14	N.S.	N.S.	N.S.
Triglyceride (mg/dL)	107.6 ± 42.0	94.5 ± 27.7	95.9 ± 27.0	91.2 ± 59.5	N.S.	N.S.	N.S.
Total cholesterol (mg/dL)	182.6 ± 54.9	134.0 ± 35.3 *	147.2 ± 44.6	132.5 ± 15.6	N.S.	0.025	N.S.
HDL-C (mg/dL)	25.9 ± 5.8	19.1 ± 5.7 *	25.9 ± 10.7	26.5 ± 8.8	N.S.	N.S.	N.S.
VLDL-C + LDL-C (mg/dL)	156.4 ± 54.3	108.9 ± 34.4 *	125.9 ± 45.2	105.4 ± 18.9	N.S.	0.021	N.S.
Total cholesterol/HDL-C (mg/dL)	7.32 ± 2.84	7.32 ± 2.85	5.89 ± 2.40	4.78 ± 1.25	0.031	N.S.	N.S.
HDL-C/VLDL-C + LDL-C	0.28 ± 0.10	0.24 ± 0.14	0.30 ± 0.18	0.36 ± 0.14	N.S.	N.S.	N.S.

Results are expressed as mean ± standard deviation for 8–10 rats per group. * *p* < 0.05 compared with the respective control via the unpaired *t*-test. ^1^ DM stands for diabetes mellitus; MBW stands for mung bean water; DM × MBW represents the diabetes and mung bean water interaction assessed by two-way ANOVA. ^2^ N.S. stands for not significant.

**Table 4:** Hepatic levels of lipids and enzymes in rats supplemented with MBW for 8 weeks.

	DM		Normal		Two Way ANOVA ^1^		
	Water	MBW	Water	MBW	DM	MBW	DM × MBW
Cholesterol (mg/g liver)	114.4 ± 24.0	100.1 ± 16.3	72.4 ± 14.2	60.6 ± 14.7	0.000	0.043	N.S.
Cholesterol (g/liver)	4.28 ± 1.30	3.75 ± 0.89	2.31 ± 0.53	1.97 ± 0.54	0.000	N.S.	N.S.
Triglyceride (mg/g liver)	76.3 ± 16.3	72.2 ± 16.7	63.7 ± 13.4	44.8 ± 9.4 *	0.000	0.021	N.S.
Triglyceride (g/liver)	2.96 ± 0.96	2.62 ± 0.85	2.12 ± 0.67	1.43 ± 0.46 *	0.000	N.S.	N.S.
Hexokinase (nmol/min/mg protein)	42.9 ± 22.2	54.4 ± 14.6	54.1 ± 33.1	63.1 ± 33.0	N.S. ^2^	N.S.	N.S.
(mmol/min/g liver)	0.22 ± 0.06	0.31 ± 0.09 *	0.28 ± 0.05	0.25 ± 0.08	N.S.	N.S.	0.028
(mmol/min/total liver)	8.37 ± 2.86	11.2 ± 3.60 *	8.86 ± 1.54	7.97 ± 2.33	N.S.	N.S.	0.049
G-6-Pase (nmol/min/mg protein)	69.9 ± 27.5	66.1 ± 25.4	60.5 ± 20.2	60.9 ± 14.5	N.S.	N.S.	N.S.
(μmol/min/g liver)	53.3 ± 21.0	50.4 ± 19.3	46.2 ± 15.4	46.4 ± 11.1	N.S.	N.S.	N.S.
(mmol/min/total liver)	2.00 ± 0.89	1.87 ± 0.81	1.47 ± 0.56	1.57 ± 0.43	N.S.	N.S.	N.S.
Hexokinase/G-6-Pase ^3^	0.61 ± 025.	0.63 ± 0.25	0.58 ± 0.17	0.60 ± 0.26	N.S.	N.S.	N.S.
G-6-P DeHase (nmol/min/mg protein)	40.7 ± 17.6	23.3 ± 12.3 *	29.8 ± 10.7	30.8 ± 8.0	N.S.	N.S.	0.046
(mmol/min/g liver)	0.34 ± 0.13	0.21 ± 0.07 *	0.27 ± 0.10	0.32 ± 0.10	N.S.	N.S.	0.018
(mmol/min/total liver)	12.5 ± 4.87	7.72 ± 2.64 *	9.03 ± 3.81	10.5 ± 4.29	N.S.	N.S.	0.031

Results are expressed as mean ± standard deviation for 8–10 rats per group. * *p* < 0.05 compared with the respective control via the unpaired *t*-test. ^1^ DM stands for diabetes mellitus; MBW stands for mung bean water; DM × MBW represents the diabetes and mung bean water interaction assessed by two-way ANOVA. ^2^ N.S. stands for not significant. ^3^ Hexokinase/G-6-Pase was calculated as the value of hexokinase (nmol/min/mg protein)/the value of G-6-Pase (nmol/min/mg protein).

**Table 5:** Fecal levels of lipids for rats supplemented with MBW for 8 weeks.

	DM		Normal		Two Way ANOVA ^1^		
	Water	MBW	Water	MBW	DM	MBW	DM × MBW
Cholesterol (mg/g) Cholesterol (mg/total feces)	11.7 ± 2.06 24.5 ± 4.78	8.10 ± 3.33 * 18.1 ± 4.09 *	10.7 ± 1.64 21.1 ± 3.79	10.2 ± 2.04 21.3 ± 7.17	N.S. ^2^ N.S.	0.019 N.S.	0.089 N.S.
Triglyceride (mg/g) Triglyceride (mg/total feces)	3.16 ± 1.41 6.68 ± 3.10	3.34 ± 1.08 6.76 ± 2.58	3.12 ± 1.86 6.18 ± 4.19	3.48 ± 1.15 7.30 ± 3.36	N.S. N.S.	N.S. N.S.	N.S. N.S.

Results are expressed as mean ± standard deviation for 8–10 rats per group. * *p* < 0.05 compared with the respective control via the unpaired *t*-test. ^1^ DM stands for diabetes mellitus; MBW stands for mung bean water; DM × MBW represents the diabetes and mung bean water interaction assessed by two-way ANOVA. ^2^ N.S. stands for not significant.

**Table 6:** Levels of hepatic antioxidant enzymes and thiobarbituric acid reactive substances (TBARSs) in tissues of rats supplemented with MBW for 8 weeks.

	DM		Normal		Two Way ANOVA ^1^		
	Water	MBW	Water	MBW	DM	MBW	DM × MBW
GSH (μmol/g liver)	28.3 ± 5.5	32.9 ± 13.2	39.7 ± 10.2	40.4 ± 11.4	0.010	N.S.	N.S.
GSSG (μmol/g liver)	9.61 ± 2.64	10.6 ± 5.27	9.25 ± 2.67	9.37 ± 3.77	N.S. ^2^	N.S.	N.S.
GSH Px (μmol NADPH decrease/min/mg protein)	0.23 ± 0.03	0.20 ± 0.01 *	0.22 ± 0.03	0.24 ± 0.03	N.S.	N.S.	0.014
(μmol NADPH decrease/min/g liver)	5.63 ± 1.19	4.60 ± 0.52 *	5.28 ± 1.23	5.93 ± 0.89	N.S.	N.S.	0.020
(mmol NADPH decrease/min/total liver)	0.21 ± 0.05	0.17 ± 0.03 *	0.17 ± 0.04	0.19 ± 0.03	N.S.	N.S.	0.038
Plasma TBARS (nmol/mL)	2.53 ± 0.40	2.10 ± 0.30 *	2.38 ± 0.39	1.99 ± 0.27 *	N.S.	N.S.	0.001
Hepatic TBARS (nmol/g tissue)	21.6 ± 9.3	11.5 ± 8.4 *	18.4 ± 9.7	21.9 ± 12.5	N.S.	N.S.	N.S.
Nephrotic TBARS (nmol/g tissue)	95.9 ± 30.3	97.6 ± 28.9	29.4 ± 9.5	34.9 ± 16.7	0.000	N.S.	N.S.
AST (U/L)	65.1 ± 26.2	81.2 ± 29.1	67. 6 ± 28.9	90.1 ± 31.9	N.S.	N.S.	N.S.
ALT (U/L)	34.5 ± 17.9	41.8 ± 15.7	32.9 ± 18.1	35.3 ± 7.0	N.S.	N.S.	N.S.

Results are expressed as mean ± standard deviation for 8–10 rats per group. * *p* < 0.05 compared with the respective control via the unpaired *t*-test. ^1^ DM stands for diabetes mellitus; MBW stands for mung bean water; DM × MBW represents the diabetes and mung bean water interaction assessed by two-way ANOVA. ^2^ N.S. stands for not significant.

## Data Availability

The original contributions presented in the study are included in this article.

## References

[1] Jagannathan R., Neves J.S., Dorcely B., Chung S.T., Tamura K., Rhee M., Bergman M. (2020). The oral glucose tolerance test: 100 years later. Diabetes Metab. Syndr. Obes..

[2] Lenzen S. (2008). The mechanisms of alloxan-and streptozotocin-induced diabetes. Diabetologia.

[3] Masiello P. (2006). Animal models of type 2 diabetes with reduced pancreatic β-cell mass. Int. J. Biochem. Cell Biol..

[4] Eleazu C.O., Eleazu K.C., Chukwuma S., Essien U.N. (2013). Review of the mechanism of cell death resulting from streptozotocin challenge in experimental animals, its practical use and potential risk to humans. J. Diabetes Metab. Disord..

[5] Ganesan K., Xu B. (2017). Polyphenol-rich lentils and their health promoting effects. Int. J. Mol. Sci..

[6] Hou D., Zhao Q., Yousaf L., Xue Y., Shen Q. (2020). Whole mung bean (*Vigna radiata* L.) supplementation prevents high-fat diet-induced obesity and disorders in a lipid profile and modulates gut microbiota in mice. Eur. J. Nutr..

[7] Hou D., Yousaf L., Xue Y., Hu J., Wu J., Hu X., Feng N., Shen Q. (2019). Mung bean (*Vigna radiata* L.): Bioactive polyphenols, polysaccharides, peptides, and health benefits. Nutrients.

[8] Diao J., Miao X., Chen H. (2022). Anti-inflammatory effects of mung bean protein hydrolysate on the lipopolysaccharide-induced RAW264. 7 macrophages. Food Sci. Biotechnol..

[9] Yao Y., Chen F., Wang M., Wang J., Ren G. (2008). Antidiabetic activity of Mung bean extracts in diabetic KK-Ay mice. J. Agric. Food Chem..

[10] Kohno M., Sugano H., Shigihara Y., Shiraishi Y., Motoyama T. (2018). Improvement of glucose and lipid metabolism via mung bean protein consumption: Clinical trials of GLUCODIA™ isolated mung bean protein in the USA and Canada. J. Nutr. Sci..

[11] Kang I., Choi S., Ha T.J., Choi M., Wi H.-R., Lee B.W., Lee M. (2015). Effects of mung bean (*Vigna radiata* L.) ethanol extracts decrease proinflammatory cytokine-induced lipogenesis in the KK-Ay diabese mouse model. J. Med. Food.

[12] Mohd Ali N., Mohd Yusof H., Long K., Yeap S.K., Ho W.Y., Beh B.K., Koh S.P., Abdullah M.P., Alitheen N.B. (2013). Antioxidant and hepatoprotective effect of aqueous extract of germinated and fermented mung bean on ethanol—mediated liver damage. BioMed Res. Int..

[13] Liu T., Yu X.H., Gao E.Z., Liu X.N., Sun L.J., Li H.L., Wang P., Zhao Y.L., Yu Z.G. (2014). Hepatoprotective effect of active constituents isolated from mung beans (*Phaseolus radiatus* L.) in an alcohol-induced liver injury mouse model. J. Food Biochem..

[14] Yang Q.-Q., Ge Y.-Y., Gunaratne A., Kong K.-W., Li H.-B., Gul K., Kumara K., Arachchi L.V., Zhu F., Corke H. (2020). Phenolic profiles, antioxidant activities, and antiproliferative activities of different mung bean (*Vigna radiata*) varieties from Sri Lanka. Food Biosci..

[15] Sehrawat N., Yadav M., Kumar S., Upadhyay S.K., Singh M., Sharma A.K. (2020). Review on health promoting biological activities of mungbean: A potent functional food of medicinal importance. Plant Arch..

[16] Listyawati S., Herawati E., Widiyani T. (2023). The Influence of Mung Bean And Ginger Extracts Combination on Blood Glucose Levels of Type-2 Diabetes Mellitus Rats Model. Proceedings of the 3rd International Conference on Biology, Science and Education (IcoBioSE 2021).

[17] Li L., Tian Y., Zhang S., Feng Y., Wang H., Cheng X., Ma Y., Zhang R., Wang C. (2022). Regulatory effect of mung bean peptide on prediabetic mice induced by high-fat diet. Front. Nutr..

[18] Li L., Tian Y., Feng Y., Zhang S., Jiang Y., Zhang Y., Zhan Y., Wang C. (2022). Improvement in mung bean peptide on high-fat diet-induced insulin resistance mice using untargeted serum metabolomics. Front. Nutr..

[19] Saeting O., Chandarajoti K., Phongphisutthinan A., Hongsprabhas P., Sae-Tan S. (2021). Water extract of mungbean (*Vigna radiata* L.) inhibits protein tyrosine phosphatase-1B in insulin-resistant HepG2 cells. Molecules.

[20] Shen X., Jiang X., Qian L., Zhang A., Zuo F., Zhang D. (2022). Polyphenol extracts from germinated mung beans can improve type 2 diabetes in mice by regulating intestinal microflora and inhibiting inflammation. Front. Nutr..

[21] Slinkard K., Singleton V.L. (1977). Total phenol analysis: Automation and comparison with manual methods. Am. J. Enol. Vitic..

[22] Chemists A. (2004). Association of Official Analytical Chemists (AOAC) Official Methods of Analysis.

[23] Allison D.B., Paultre F., Maggio C., Mezzitis N., Pi-Sunyer F.X. (1995). The use of areas under curves in diabetes research. Diabetes Care.

[24] Takehisa F., Suzuki Y. (1990). Effect of guar gum and cholestyramine on plasma lipoprotein cholesterol in rats. J. Jpn. Soc. Nutr. Food Sci..

[25] Folch J., Lees M., Stanley G.S. (1957). A simple method for the isolation and purification of total lipides from animal tissues. J. Biol. Chem..

[26] Griffith O.W., Meister A. (1985). Origin and turnover of mitochondrial glutathione. Proc. Natl. Acad. Sci. USA.

[27] Erickson R.H., Zakim D., Vessey D.A. (1978). Preparation and properties of a phospholipid-free form of microsomal UDP-glucuronyltransferase. Biochemistry.

[28] Matthews D.R., Hosker J.P., Rudenski A.S., Naylor B., Treacher D.F., Turner R.C. (1985). Homeostasis model assessment: Insulin resistance and β-cell function from fasting plasma glucose and insulin concentrations in man. Diabetologia.

[29] Pari L., Satheesh M.A. (2006). Effect of pterostilbene on hepatic key enzymes of glucose metabolism in streptozotocin-and nicotinamide-induced diabetic rats. Life Sci..

[30] Masiello P., Broca C., Gross R., Roye M., Manteghetti M., Hillaire-Buys D., Novelli M., Ribes G. (1998). Experimental NIDDM: Development of a new model in adult rats administered streptozotocin and nicotinamide. Diabetes.

[31] Al-Khnifsawi M., Al-Rasadi K., Santos R.D., Foy K.A. (2021). From the President of the International Atherosclerosis Society: The Iraqi Lipid Clinics Network. J. Clin. Lipidol..

[32] Massillon D., Barzilai N., Chen W., Hu M., Rossetti L. (1996). Glucose Regulates in Vivo Glucose-6-phosphatase Gene Expression in the Liver of Diabetic Rats (∗). J. Biol. Chem..

[33] Sies H. (1999). Glutathione and its role in cellular functions. Free Radic. Biol. Med..

[34] Esterbauer H., Cheeseman K. (1990). Determination of aldehydic lipid peroxidation products: Malonaldehyde and 4-hydroxynonenal. Methods Enzymol..

[35] Prati D., Taioli E., Zanella A., Torre E.D., Butelli S., Del Vecchio E., Vianello L., Zanuso F., Mozzi F., Milani S. (2002). Updated definitions of healthy ranges for serum alanine aminotransferase levels. Ann. Intern. Med..

[36] Grover J., Yadav S., Vats V. (2002). Medicinal plants of India with anti-diabetic potential. J. Ethnopharmacol..

[37] Adams L.A., Angulo P., Lindor K.D. (2005). Nonalcoholic fatty liver disease. Can. Med. Assoc. J..

[38] Lee K.-G., Shibamoto T. (2000). Antioxidant properties of aroma compounds isolated from soybeans and mung beans. J. Agric. Food Chem..

[39] Kamalakkannan N., Prince P.S.M. (2006). Rutin improves the antioxidant status in streptozotocin-induced diabetic rat tissues. Mol. Cell. Biochem..

[40] Lee J.-S. (2006). Effects of soy protein and genistein on blood glucose, antioxidant enzyme activities, and lipid profile in streptozotocin-induced diabetic rats. Life Sci..

[41] Manco M., Calvani M., Mingrone G. (2004). Effects of dietary fatty acids on insulin sensitivity and secretion. Diabetes Obes. Metab..

[42] Park S.A., Choi M.-S., Cho S.-Y., Seo J.-S., Jung U.J., Kim M.-J., Sung M.-K., Park Y.B., Lee M.-K. (2006). Genistein and daidzein modulate hepatic glucose and lipid regulating enzyme activities in C57BL/KsJ-db/db mice. Life Sci..

[43] Prince P.S.M., Kamalakkannan N. (2006). Rutin improves glucose homeostasis in streptozotocin diabetic tissues by altering glycolytic and gluconeogenic enzymes. J. Biochem. Mol. Toxicol..

[44] Coskun O., Kanter M., Korkmaz A., Oter S. (2005). Quercetin, a flavonoid antioxidant, prevents and protects streptozotocin-induced oxidative stress and β-cell damage in rat pancreas. Pharmacol. Res..

